# Household food security contextualised: A comparison of Ambros and Maramanzhi villages, South Africa

**DOI:** 10.1016/j.heliyon.2024.e39971

**Published:** 2024-10-30

**Authors:** Felicity Aphiwe Mkhongi, Walter Musakwa, Tholang Mokhele

**Affiliations:** a Department of Geography, Environmental Management and Energy Studies, University of Johannesburg, Auckland Park, Johannesburg, 2093, South Africa; b Geospatial Analytics Unit, eResearch Knowledge Centre, Human Sciences Research Council, Pretoria, 0002, South Africa

**Keywords:** Cultivation, Deagrarianization, Food security, Rural households, Smallholder farmers, Sustainable development goal (SDG) 2

## Abstract

Smallholder agriculture plays a crucial role in achieving food security, particularly at the household level. However, fallow fields are progressively increasing in former homelands of South Africa. While substantial efforts have been devoted towards addressing food insecurity, access to arable land has not translated to sustainable crop production for smallholder farmers in former homelands of the country. This paper analyses household food security in the context of deagrarianization in two villages, Ambros (Eastern Cape) and Maramanzhi (Limpopo). Using a mixed-method approach, a total of 106 semi-structured questionnaires were administered through face-to-face interviews with household heads. Descriptive statistics were analysed using the IBM SPSS Statistics 27.0 computer program. Meanwhile, qualitative data was coded and content analysis was conducted on NVivo 12 software. The key findings revealed that the primary household income in the study area was earned from social grants. Furthermore, home gardens, with an average size of 4100m2 in Ambros and 4400m2 in Maramanzhi village, played a crucial role in supporting household crop production. However, food insecurity threatened the sustainability of rural livelihoods because the Household Food Insecurity Access Scale (HFIAS) indicator highlighted that a majority of 54% of households in Ambros and 69% in Maramanzhi were mildly food insecure. Food insecurity challenges varied between the two villages but these were mainly perpetuated by food shortages caused by insufficient monthly income and waning household crop production. Although acquiring food was rated as a very important reason for cultivating in both villages, deagrarianization eroded opportunities for increased crop production. Among other solutions, this study recommends that the government improve the delivery of basic services such as water to promote household crop production and the revitalization of fallow fields. These transformations can potentially enhance food security, income and employment opportunities for rural households, contributing to the sustainability of rural livelihoods.

## Highlights

• Crop production can enhance rural household food security.
• Deagrarianization threatens household food security.
• Low income affects food access for rural households.
• The degree of food access challenges varies among households.
• Rural households mainly cultivate to improve food availability.

## 1 Introduction

Achieving food security is a critical focus of the 2030 Agenda for Sustainable Development (2030 AfSD) and Agenda 2063-The Africa we Want [1]. The 2030 AfSD – Sustainable Development Goal (SDG) 2 seeks to end hunger, achieve food security and improved nutrition, and promote sustainable agriculture [1]. Despite these goals, food insecurity remains an immense challenge globally, as an estimated 28.9% of the global population was moderately or severely food insecure in 2023 [2]. Among various causes, the high levels of food insecurity in rural households of South Africa have been attributed to low agricultural productivity and a decline in field cultivation [[3], [4], [5]]. These agrarian changes point to deagrarianization, a phenomenon that has negative socio-economic impacts, threatening national and local food security, rural livelihoods and landscapes [[6], [7], [8]]. Although the decline in the area of cultivated fields reduces the diversity and quality of diets, home gardens have played an important role in achieving household food security [[9], [10], [11]]. There is consensus that smallholder agriculture plays a fundamental role in improving livelihoods and reducing food insecurity vulnerabilities, particularly in sub-Saharan Africa [[12], [13], [14], [15]]. This implies that interventions to ensure sustainable crop production and food security for rural households require more than just access to land, but also the revitalization of fallow fields. Studies related to food security and deagrarianization in South Africa are linked with contextualising the contributions of the retail sector in rural food security [16], analysing garden production [11], analysing agrarian land use changes [5,17] and the effects of cash transfers on rural livelihoods [18]. Smallholder farmers in former homelands of South Africa have access to fields but have progressively disengaged from cultivation, despite household food insecurity challenges in the country [5,19,20]. Thus, the researcher identified a knowledge gap concerning studies that assess the impacts of deagrarianization on household food security. Therefore, this paper aims to analyse household food security in two villages, namely, Ambros, located in Umzimvubu Local Municipality, Eastern Cape and Maramanzhi, situated in Musina Local Municipality, Limpopo. Our analysis places at its centre the following objectives.

1. To examine access to arable land.
2. To determine food availability.
3. To evaluate food access and utilization.

### 1.1 Food (In)security contextualised

Food security is a multi-dimensional concept which has evolved since the early 1940s [21,22]. The revised definition explains that food security refers to access for all people at all times to enough food for an active, healthy life [23]. Food security exists when all people, at all times, have physical and economic access to sufficient, safe and nutritious food that meets their dietary needs and food preferences for an active and healthy life [23]. Achieving food security requires four main pillars of the concept, namely, availability, access, utilization and stability to be fulfilled (Table 1) [24].

**Table 1:** Pillars of food security, Source [24].

Pillar	Description
Availability	*“The availability of sufficient quantities of food of appropriate quality, supplied through domestic production or imports (including food aid)”.*
Access	*“Access by individuals to adequate resources (entitlements) for acquiring appropriate foods for a nutritious diet”.*
Utilization	*“The utilization of food through adequate diet, clean water, sanitation and health care to reach a state of nutritional well-being where all physiological needs are met”.*
Stability	*“To be food secure, a population, household or individual must have access to adequate food at all times”.*

In order to understand the dynamics of food security in sub-Saharan Africa, researchers have analysed household food security in the context of various factors. These include gender-based access to education and employment [25], the role of gender in accessing food [26] and migration trends [27]. Other factors include land ownership patterns and the role of women in farming [28], government agricultural development support [29], organic agriculture [30] and market participation of indigenous smallholder farmers [31]. South Africa is not immune to hunger and food insecurity. However, the country's government continues to implement policies, programmes and intervention measures to address this vulnerability. Food security is a constitutional right enshrined in Section 27 of the Constitution of South Africa which indicates that everyone has the right to have access to sufficient food and water [32]. The government has a responsibility to use reasonable legislative and other measures to ensure the right is achieved. Additionally, household food insecurity results from an inability to meet daily food requirements and anxiety about the ability to produce and/or access food in the future [33]. The status of food (in)security for a person or household is not constant but changes over time [34]. At the household level, households are food insecure if they lack adequate food to maintain an active and healthy lifestyle for all of their members [35,36].

Various indicators have been adopted to assess, analyse and provide empirical evidence that quantifies food security in South Africa. Indicators of food security evaluate key components of the phenomenon, including contributing factors, education, mortality as well as nutrition [37]. Some surveys such as the Community Survey (CS), General Household Survey (GHS), Income and Expenditure Survey (IES), Labour Force Survey (LFS), National Food Consumption Survey (NFCS) and the South African Social Attitudes Survey (SASAS) include indicators that inform about the different pillars of food security in South Africa [38]. Recently, the South African government made significant investments aimed at improving the measurement of food security. Subsequently, the Food Insecurity Experience Scale (FIES) module was introduced to the General Household Survey in 2019 [39]. Studies that have applied food security indicators discovered that South Africa is food secure at the national level but numerous households remain food insecure [4,35,[40], [41], [42], [43], [44], [45]]. Although there has been a decrease from 2002, 13.5% of households in South Africa were vulnerable to hunger in 2023 [46].

Table 2 highlights the prevalence of food access challenges in the Eastern Cape and Limpopo provinces in South Africa. This was measured through a set of questions from the Household Food Insecurity Access Scale (HFIAS) which has a 30-day recall period. Despite the majority of households in Eastern Cape (73.5%) and Limpopo (93.1%) indicating adequate access to food, food insecurity remains a challenge as households continue to experience inadequate and severely inadequate food access [46].

**Table 2:** Household food access distribution in 2023, Source [46].

	Eastern Cape	Limpopo
Severely inadequate food access	9.1 %	1.2 %
Inadequate food access	17.4 %	5.7 %
Adequate food access	73.5 %	93.1 %

### 1.2 Smallholder agriculture in enhancing household food security

The South African agricultural sector comprises a dual system defined by (i) capital-intensive and well-developed commercial agriculture, which contributes to national food security, and (ii) the less-resourced and less-developed smallholder agriculture practiced by smallholder farmers that farm primarily for household consumption [47,48]. Commercial agriculture is practiced in formerly white-owned farmlands, but smallholder agriculture is common in former homelands of the country [49]. Despite resource disparities, Limpopo, with 38.2%, followed by Mpumalanga (33.1%) and Eastern Cape (32.7%) provinces, had the highest percentage of households engaged in agricultural activities in South Africa during the year 2023 [46]. Accounting for the main reasons for agricultural engagement, the vast majority of households in the country (75.5%) were involved to secure an additional source of food, while 11.7% were engaged to acquire their main source of food [46]. This distribution emphasizes the significant contributions of agricultural engagements in achieving household food demands in South Africa [42,[50], [51], [52]].

A growing body of knowledge suggests that part of the solution for addressing local-level food insecurity in South Africa is encouraging rural households to actively engage in crop production [53,54]. The role of smallholder agriculture in alleviating food insecurity and poverty remains ambiguous and highly contested. However, in contrast to non-farming households, farming households in the Eastern Cape are significantly correlated with lower levels of hunger [55]. Smallholder agriculture not only increases food availability and diversifies diets [50,56], but it also supplements and delivers an additional source of food [12,47]. Moreover, it guarantees household food and nutrition security [13,44,57,58] and creates a source of employment [59]. As more people disengage from cultivation, household food security becomes compromised. This reorientation of livelihoods in sub-Saharan Africa not only creates opportunities for food prices to increase but also increases the reliance on purchased food for the already income-constrained rural poor [16].

### 1.3 Conceptual framework

Empirical evidence suggests that household crop production is an effective solution for alleviating household food insecurity and poverty in South Africa and other developing countries [[60], [61], [62]]. Attempts to comprehend the potential contribution of agricultural development in rural poverty reduction should place farming in a wider livelihood context [63]. Hence, this study adopted a sustainable livelihoods approach for data collection and analysis. Livelihoods research emerged to concentrate on poverty-related research, with a particular focus on examining rural livelihoods [64].

**Fig. 1:**
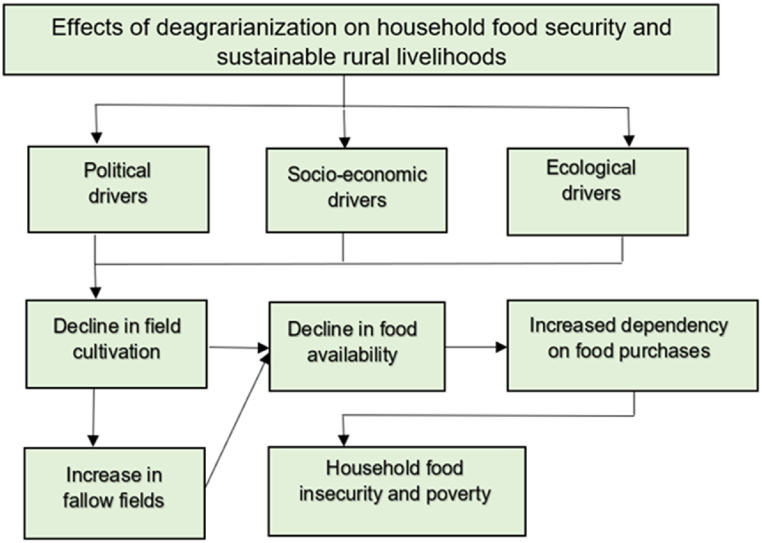
Conceptual framework.

The impacts of deagrarianization on household food security and sustainable rural livelihoods are depicted in Fig. 1. Due to various political, socio-economic and ecological drivers of deagrarianization, smallholder farmers in South African former homelands have progressively disengaged from field cultivation. A decline in the area of cultivated fields reduces available food for rural households, leading to more reliance on purchased food. This reliance increases food insecurity vulnerabilities and negatively affects the sustainability of rural livelihoods, mainly because the majority of households depend on social grants, despite the high cost of living in South Africa [65]. Thus, this approach allowed for a comprehensive understanding of the availability, access and utilization pillars of food security in the context of deagrarianization in the study area.

## 2 Materials and methods

### 2.1 Description of study area

The study was conducted in two villages representing different geographical and socio-economic contexts of South Africa. The first village, Ambros is situated within Umzimvubu Local Municipality, Alfred Nzo District Municipality, Eastern Cape. The majority of land within this local municipality is covered by dispersed, low-density traditional settlements [66]. Land cover classifications indicate that cultivated subsistence land covers 30 672 ha (12.22%) of the total land area of Umzimvubu Local Municipality [66]. Covering an area of 2.27 square kilometres, Ambros has 152 households with a total population of 566 [67]. The nearest towns that provide amenities for this village are Mount Frere (47.9km away) and Matatiele (68.4km away). The second village, Maramanzhi, is situated within Musina Local Municipality, Vhembe District Municipality, Limpopo. This village covers an area of 1.15 square kilometres and has 171 households with a population of 664 [67]. The main towns used by locals are Thohoyandou (86.5km away) and Musina (113.5km away). The socio-economic characteristics of Ambros and Maramanzhi are summarised in Table 3 below.

**Table 3:** General characteristics of the two study villages.

Village Characteristics	Ambros (Eastern Cape)	Maramanzhi (Limpopo)
Dominant language	IsiXhosa	Tshivenda
Distance to the nearest town	Approximately 48 km	Approximately 87 km
Market access	Limited	Very easy
Road surface	Gravel	Gravel and tar
Main source of household drinking water	River	Household taps (in the yard)
Main source of household electricity	Solar energy	In-house conventional and prepaid meter

### 2.2 Data collection

Primary data used in this study was collected in August 2021. This data were analysed using a mixed approach of both qualitative and quantitative methods. Semi-structured questionnaires were used to facilitate face-to-face interviews with household heads. A probability sampling design was adopted, and simple random sampling was used to select these household heads. This selection was based on the accessibility of each household and the availability and willingness of each participant household head. Subsequently, 60 semi-structured interviews were conducted in each village. However, 54 household heads in Ambros and 52 in Maramanzhi completed these interviews. This equated to a total of 106 participants for this study. The study focused on household heads from Ambros and Maramanzhi since these villages are located in provinces that have a higher prevalence of food access challenges. Smallholder farmers in Ambros and Maramanzhi villages also cultivate fields and home gardens, allowing for an in-depth comprehension of nuances inherent in crop production and, subsequently, food access, availability and utilization pillars of food security. This study was approved on August 12, 2021, by the Ethics and Plagiarism Committee (FEPC) of the Faculty of Engineering and the Built Environment at the University of Johannesburg, with ethics approval reference UJ-FEBE_FEPC_00307. Thereafter, data collection commenced on August 15, 2021. Written consent was obtained from each household head before commencing each interview. All the participants were informed that their participation was voluntary, their responses were anonymous and there was an option to withdraw at any time during the survey. The questions and terminology included in the questionnaire were translated into local languages, mainly IsiXhosa in Ambros and Tshivenda in Maramanzhi. Field assistants from Maramanzhi were trained to assist with data collection in the village, as this improved communication and the understanding of the questionnaire content. Quantitative questions included the demographics, field or home garden ownership as well as types of cultivated crops, food access dynamics and the main source of household drinking water. Qualitative questions were used to capture in-depth explanations and perceptions related to cultivation patterns and food access, availability and utilization. All responses were recorded on hardcopy questionnaires and captured on Google forms.

### 2.3 Data analysis

All responses were downloaded from Google Forms and saved as a Microsoft Excel spreadsheet. Qualitative data, such as explanations for crop preferences, was analysed on NVivo 12 software. The data were coded and content analysis was conducted to identify similar patterns from the responses. Common terms and statements were used to form themes on perceptions linked to food availability, access and utilization. The responses were categorised to provide meaningful explanations that support the study. All quantitative data such as gender, age, field, or home garden ownership were coded to prepare for descriptive analysis on the IBM Statistical Package for Social Sciences (SPSS) Statistics 27.0 computer program. Custom tables were created to provide frequencies and percentages that summarize the results. The HFIAS formed part of the quantitative results. This indicator was applied to measure the extent of food access challenges experienced by households in the study area. The scale is a self-reported food insecurity measure based on a methodology developed by the United States Agency for International Development (USAID) in the Food and Nutrition Technical Assistance (FANTA) Project [37]. HFIAS covers nine questions related to the frequency of certain conditions that signify challenges in accessing food at a given household, within a 30-day recall period. A total score was derived from the questions to determine the severity of food access challenges. Each household can get a minimum score of 0, while the maximum cannot exceed 27. The lower the score, the fewer food access challenges experienced in a household and the higher the score, the more food access challenges experienced in a household [37]. The score for each household was calculated by tallying the coded frequency for each of the nine frequency-of-occurrence questions. Each occurrence question was coded as zero for instances where the answer was “no”. In the case where the answer to an occurrence question was “yes”, a frequency-of-occurrence question was posed. The responses were coded as “one” if a condition rarely happened (once or twice), “two” if a condition sometimes occurred (three to ten times), or “three” if a condition often occurred (more than ten times) in the past 30 days [37]. Thereafter, the average HFIAS score was calculated by dividing the sum of HFIAS scores for each village by the number of sampled households (in each village) [37]. This assisted with categorizing the prevalence of household food access challenges into four levels of food (in) security status, namely, food secure, mildly food insecure, moderately food insecure and severely food insecure. A household was regarded as food secure if the HFIAS was less than or equal to 1, mildly food insecure if the HFIAS was between 2 and 8, moderately food insecure if the HFIAS was between 9 and 17 and severely food insecure if the HFIAS was greater than or equal to 18 [37]. This means a household was regarded as food secure if it experienced none of the food access conditions and rarely experienced worry about not having enough food. A mildly food insecure household worried about not having enough food (either sometimes or often). A moderately food insecure household sacrificed quality more frequently by sometimes or often eating a monotonous diet or undesirable foods. A severely food-insecure household reduced meal sizes or the number of meals often and also experienced some of the three most severe conditions, which are running out of food, going to bed hungry, or going a whole day and night without eating [37].

In terms of spatial analysis for maize productivity, data titled GGCP10: A Global Gridded Crop Production Dataset at 10 km Resolution from 2010 to 2020 was acquired from Harvard Dataverse. This data were used to provide information on maize productivity during the year 2020. This year was selected to provide maize data for the last year preceding data collection of this study. ArcGIS Pro 3.0.3 version was used to show the spatial distribution of maize productivity in Eastern Cape, including Ambros village and Limpopo including Maramanzhi village. A feature class was created to define a boundary for each village. Thereafter, these boundaries were projected using the define projection option under data management tools. The coordinate system of the boundaries was projected to the WGS 1984 World Mercator. The clip tool was used to extract the layer for maize productivity in the two study provinces (Eastern Cape and Limpopo). Subsequently, the layer for each village boundary was added to focus on village-level maize productivity. A map showing the spatial distribution of maize productivity in Ambros and Maramanzhi villages was created on ArcGIS Pro. Symbology was used to classify maize productivity into 5 classes per province.

## 3 Results

### 3.1 Household characteristics

Table 4 provides a summary of the socioeconomic attributes of the sampled household heads. More than half of household heads (61%) in Ambros were males, meanwhile, the majority (63%) in Maramanzhi were females. In terms of age, those aged between 40 and 49 were slightly more prevalent in Ambros (33%) than in Maramanzhi (30%). Household sizes in the study area varied but most of the households (that is, in both villages) comprised 1–3 members. While there were no households in Ambros with ten or more members, in Maramanzhi 4% of the households indicated they lived with ten or more than ten family members. Under half of the household heads in Ambros (48%) and Maramanzhi (31%) indicated secondary education to be their highest completed level of education.

**Table 4:** Socio-economic attributes of household heads.

Variable	Category	Ambros		Maramanzhi	
		Frequency (n)	Percentage (%)	Frequency (n)	Percentage (%)
Gender	Male	33	61	19	37
	Female	21	39	33	63
Age	90–99	1	2	2	4
	80–89	4	7	5	10
	70–79	10	19	8	15
	60–69	11	20	6	12
	50–59	7	13	12	23
	40–49	18	33	16	30
	30–39	3	6	3	6
Household size	1–3	29	54	22	42
	4–6	20	37	21	40
	7–9	5	9	7	14
	10+	0	0	2	4
Education	No formal education	2	4	11	21
	Primary	13	24	11	21
	Secondary	26	48	16	31
	Tertiary	13	24	14	27

Regarding income earnings per month, Fig. 2(a) illustrates that the majority (83%) of household heads in Ambros received less than R3 000, while the majority (46%) in Maramanzhi received between R3 001 and R6 000. None of the household heads in Ambros reported receiving an income between R6 001 and R9 000, but 17% received this level of income in Maramanzhi. Fig. 2(b) indicates that social grants were identified as the principal source of monthly income in both villages, namely 57% and 63%, in Ambros and Maramanzhi, respectively.

**Fig. 2(a):**
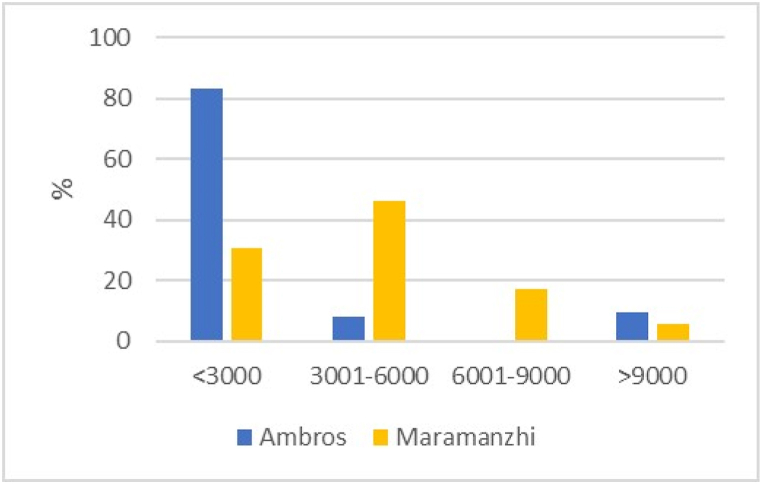
Household income per month.

**Fig. 2(b):**
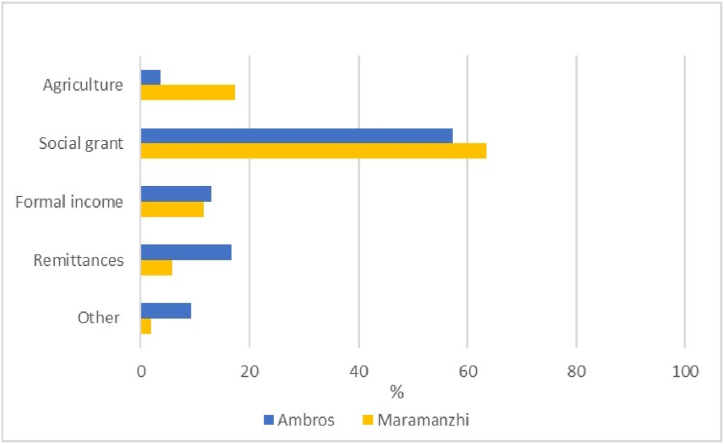
Primary household income source.

### 3.2 Food availability

**Fig. 3:**
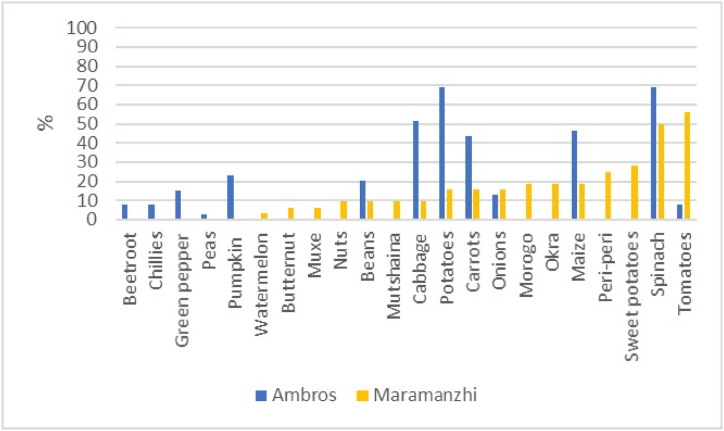
Crops cultivated on home gardens.

Fig. 3 shows the different types of crops cultivated in home gardens of the study area. The major crops reported in Ambros included potatoes (69%), spinach (69%), cabbage (51%), maize (46%) and carrots (44%). Household heads in Maramanzhi reported tomatoes (56%), spinach (50%), sweet potatoes (28%) and peri-peri (25%) to be the most cultivated crops. Regarding the frequency of purchasing food for monthly consumption, the majority of households reported purchasing food monthly in both Ambros (89%) and Maramanzhi (98%).

**Fig. 4:**
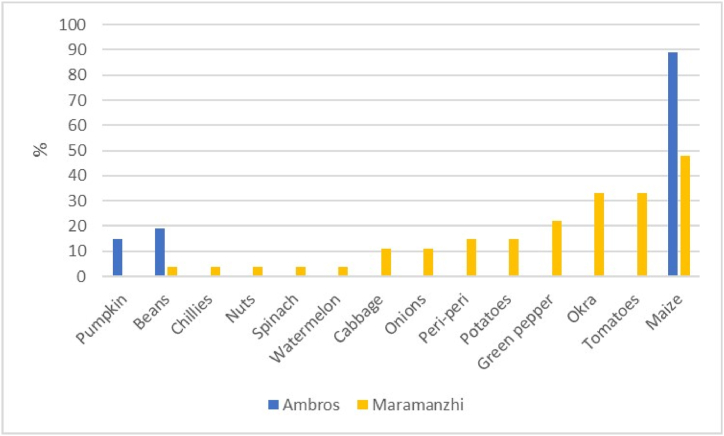
Crops cultivated on fields.

Fig. 4 presents the various types of crops cultivated in fields of the study area. Maize was the most dominant crop in Ambros (89%) and Maramanzhi (48%). Other crops reported to be cultivated in Ambros included beans (19%) and pumpkins (15%). Okra (33%), tomatoes (33%), and green pepper (22%) were also some of the reportedly grown crops in Maramanzhi.

**Fig. 5:**
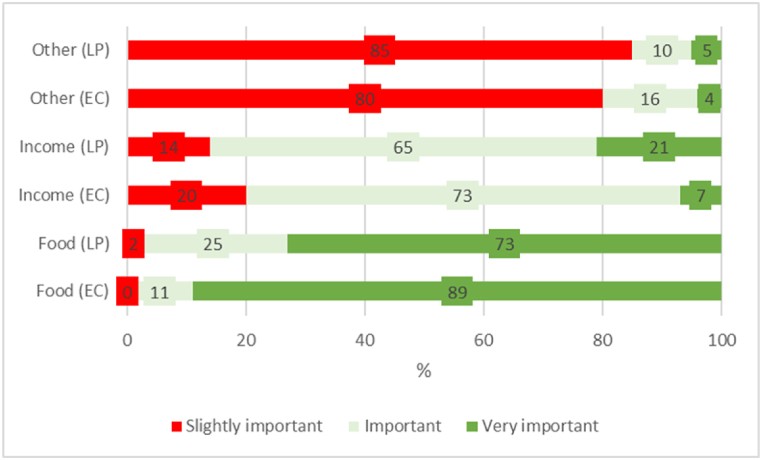
The main reason for cultivating fields and/or gardens (EC= Ambros village and LP = Maramanzhi village).

In terms of the motive for cultivation (Fig. 5), the majority of household heads in Ambros (89%) and Maramanzhi (73%) rated food as a very important reason for engaging in cultivation. Cultivating for income was mostly rated as important in both Ambros (73%) and Maramanzhi (65%). Cultivating for other reasons such as exercising, recorded the highest proportion of slightly important (80% in Ambros and 85% in Maramanzhi). Concerning access to selling produce, the majority (60%) did not have access in Ambros, while only 43% household heads did not have access in Maramanzhi.

In 2020, maize productivity in the Eastern Cape province ranged between 0.001 and 14.185 kilo tons, but Ambros village was located in a zone with productivity between 0.001 and 0.501 kilo tons (Fig. 6a). Maize productivity in Limpopo ranged between 0 and 15.741 kilo tons in 2020, but productivity in Maramanzhi village ranged between 0 and 0.617 kilo tons (Fig. 6b).

**Fig. 6(a):**
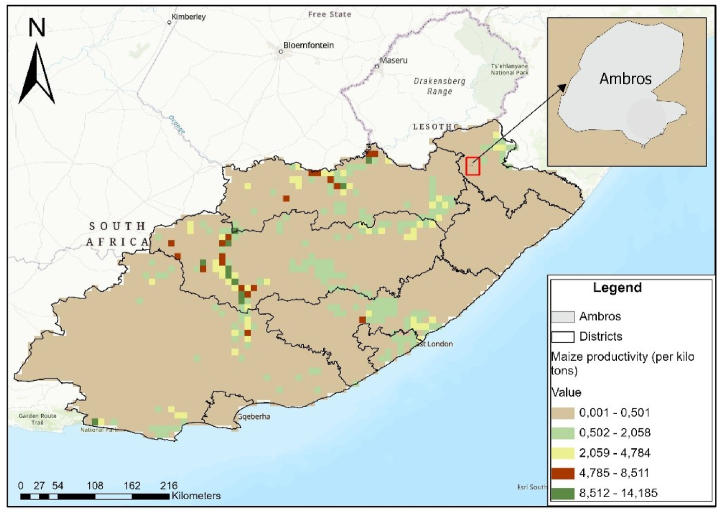
Maize productivity in Ambros (Created by the author on ArcGIS Pro, Source: Harvard Dataverse).

**Fig. 6(b):**
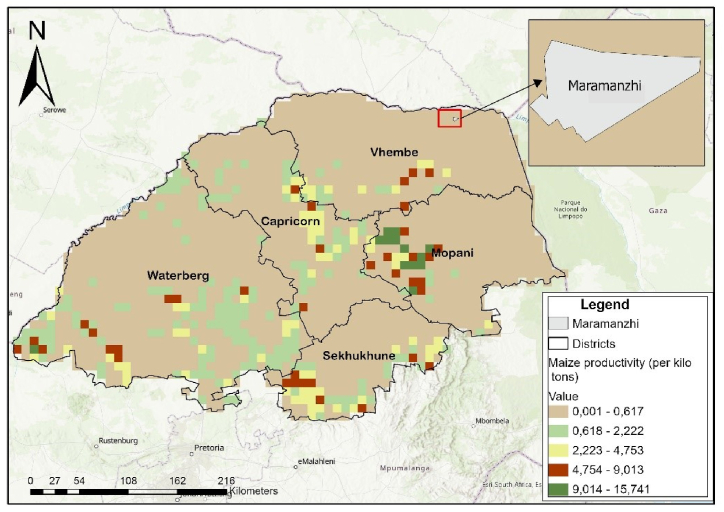
Maize productivity in Maramanzhi (Created by the author on ArcGIS Pro, Source: Harvard Dataverse).

### 3.3 Food access

Sampled households owned either a field, home garden, or both of the aforementioned (Table 5). Disaggregated by gender, a greater proportion of fields (62%) and home gardens (59%) were owned by male-headed households in Ambros. The average size of these fields was 6000m2 and home gardens were 4100m2. The majority of these fields (80%) and home gardens (95%) were last cultivated in the period 2016–2021. One respondent elaborated that:

*“Back then, during the 90s and early 2000s, life was good; I used to cultivate my whole field with maize, but now things have changed; everything needs money. I only cultivate a small plot of land. I had stopped cultivatingmy field for the past 6 years but situations were better this year (2021) because the government assisted us”* (70-year-old male respondent from Ambros village).

**Table 5:** Land ownership attributes.

Variable	Category	Ambros (%)	Maramanzhi (%)
Field ownership	Yes	48	55
Field ownership by gender	Male	62	33
	Female	38	67
Last field cultivation	Before 2010	10	0
	In the period 2011–2015	10	7
	In the period 2016–2021	80	93
Home garden ownership	Yes	72	62
Home garden ownership by gender	Male	59	44
	Female	41	56
Last home garden cultivation	Before 2010	0	0
	In the period 2011–2015	5	0
	In the period 2016–2021	95	100
Field and home garden ownership	Yes	44	27
Main smallholder farmer involved in cultivation	Father	46	52
	Mother	28	50
	Child	17	17
	Labourer	6	4

Conversely, a higher percentage of fields (67%) and home gardens (56%) were owned by female-headed households in Maramanzhi. The average size of these fields was 6200m2 and home gardens were 4400m2. The majority of these fields (93%) and home gardens (100%) were last cultivated in the period 2016–2021. One respondent elaborated that:

“*My garden is big, but it was bigger than this, now the soil quality has changed, sometimes crops die because it can be very hot in this area”* (77-year-old male respondent from Maramanzhi village).

When considering those who own both fields and home gardens, Ambros constituted 44% of households, while Maramanzhi accounted for 27% of households. A higher proportion of fathers (46% in Ambros) and 52% in Maramanzhi were the main members involved in cultivation.

Table 6 highlights food access dynamics. While some household heads reported accessing food through cultivation, a particularly high proportion (96% in Ambros and 88% in Maramanzhi) purchased household food.

**Table 6:** Food access dynamics.

Variable	Category	Ambros (%)	Maramanzhi (%)
Food access	Cultivation	70	75
	Purchasing	96	88
Monthly income enables purchasing required food for the month	Never	46	15
	Sometimes	15	39
	Often	30	19
	Always	9	27

Some of the common responses for purchasing food, as reported by household heads included:

*“We buy food my child, even though it is expensive. We have no choice because even our crops are not enough to feed us the whole month”* (Respondent from Ambros village with a household size of 4 members).

*“If things went my way I would not purchase food, instead I would just eat crops from my garden. But what can we do because we need maize and a lot of food to last us the whole month”* (Respondent from Maramanzhi village with a household size of 5 members).

Responses to whether monthly income enabled household heads to purchase all the food required for the month, varied considerably across both villages. The most reported response in Ambros was never (46%) and sometimes (39%) in Maramanzhi.

Analysis of whether cultivation reduced money spent on household food (Table 7), shows that a greater percentage (32%) of household heads in Ambros responded always, meanwhile, a greater percentage (31%) responded sometimes in Maramanzhi. About 67% of household heads reported that purchasing has made life easier in Maramanzhi, but the majority (78%) indicated purchasing has made life difficult in Ambros. More than half of the participants (69%) in Ambros and 67% in Maramanzhi indicated they experience food shortages during the month. When considering coping strategies adopted by these households, a higher proportion (49%) of households loan from the supermarket in Ambros, while more than half (54%) of households borrow money in Maramanzhi.

**Table 7:** Food perceptions and coping strategies.

Variable	Category	Ambros (%)	Maramanzhi (%)
Does cultivation reduce money spent on household food?	Never	22	27
	Sometimes	24	31
	Often	22	13
	Always	32	29
Has purchasing made life easier or difficult?	Easier	22	67
	Difficult	78	33
Do you experience food shortages during the month?	Yes	69	67
	No	31	33
Household coping strategies	Eat less food	8	46
	Borrow money	43	54
	Loan from the supermarket	49	0

**Fig. 7:**
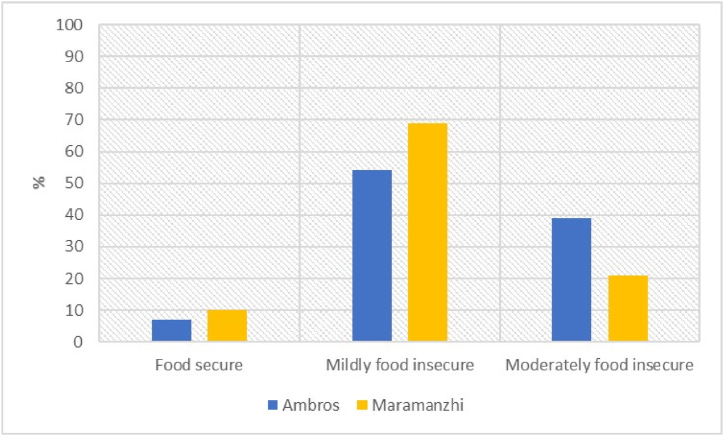
Household food security categories, using HFIAS.

Fig. 7 below shows the prevalence of food insecurity among the sampled households. The average HFIAS score for Ambros was 12.8, with a range of 0–27. Food secure households in Ambros constituted (7%), followed by mildly food insecure (54%) and moderately food insecure (39%). Results for Maramanzhi village indicated an average HFIAS score of 12.0, with a range of 0–27. Only 10% of households were food secure, 69% were mildly food insecure and 21% were moderately food insecure.

**Fig. 8:**
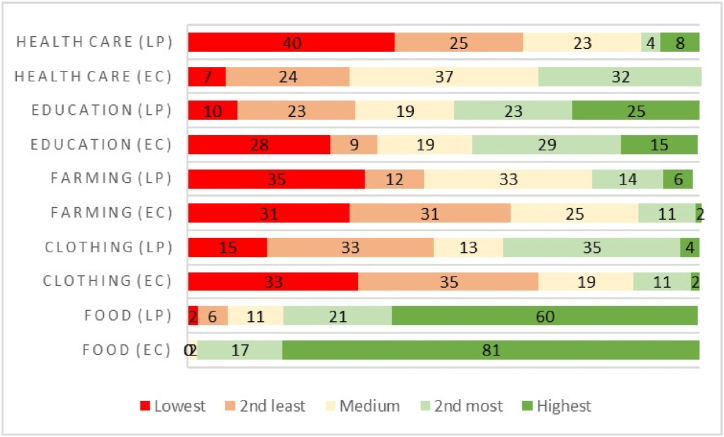
Ranking of household expenses (EC= Ambros village and LP = Maramanzhi village).

Fig. 8 depicts household expenditure on health care, education, farming, clothing and food in each village (EC= Ambros village and LP = Maramanzhi village). The proportion of household expenditures varied disproportionately in each village. Farming expenses were ranked the as the least household expense in both Ambros (31%) and Maramanzhi (35%). Household heads indicated that food accounts for the highest household expense in Ambros (81%) and Maramanzhi (60%).

### 3.4 Food utilization

**Fig. 9:**
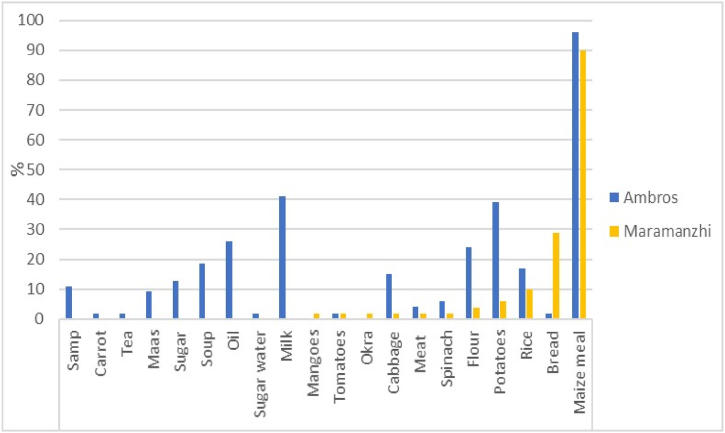
Household basic food requirement.

Fig. 9 illustrates the food considered to be a basic household requirement. Maize meal was reported as the leading food necessity in both Ambros (96%) and Maramanzhi (90%). This was followed by milk (41%) and potatoes (39%) in Ambros; bread (29%) and rice (10%) were among the leading requirements in Maramanzhi.

In terms of crop preferences (Table 8), a greater proportion of household heads in Ambros (63%) and Maramanzhi (62%) preferred cultivated rather than purchased crops. The predominant method for storing food was indicated as cupboard storage (98%) in Ambros, meanwhile, fridge storage (85%) was the most dominant method in Maramanzhi. Other food storage methods indicated in Maramanzhi included cupboards (46%), drying (8%) and containers (8%). Some of the responses from respondents were:

*“My old age grant assists me with income for buying food and other needs, but I wish I could have more money to buy seeds so I can cultivate a bigger spaceon my garden. The government also needs to assist us with water. There is no rain in this village and our taps are always dry”* (81-year-old male respondent from Ambros village).

*“I must cultivate more crops so that I can have more produce to send to the markets when the truck comes. Maybe I will have a bigger field next year*” (38-year-old female respondent from Maramanzhi village).

**Table 8:** Crop preferences and food storage methods.

Variable	Category	Ambros (%)	Maramanzhi (%)
Crop preference	Cultivated	63	62
	Purchased	37	38
Food storage methods	Containers	0	8
	Cupboard	98	46
	Digging	0	4
	Drying	0	8
	Fridge	23	85

**Fig. 10:**
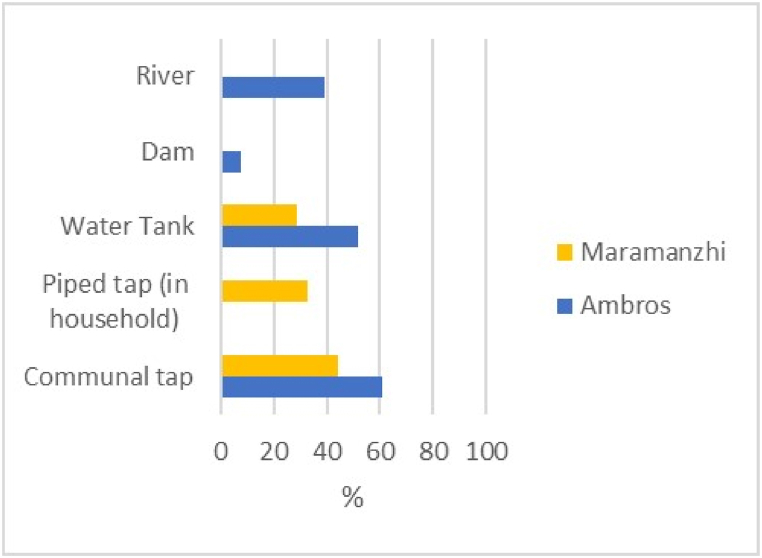
Household source of drinking water.

Fig. 10 illustrates that communal taps were indicated as the main source of drinking water in both Ambros (61%) and Maramanzhi (44%). This was followed by piped tap water within the household (33%) and water tanks (29%) in Maramanzhi village. Those who reported the use of water tanks constituted 52%, followed by water from a river (39%) and water from a dam (7%) in Ambros village.

**Fig. 11:**
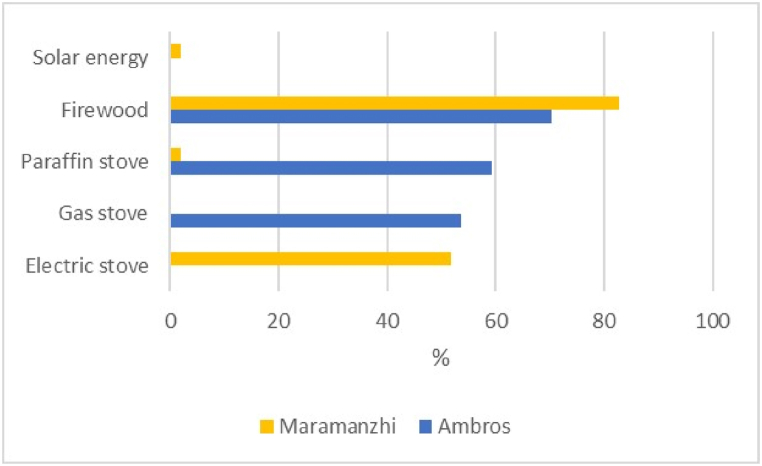
Source of energy for cooking.

Fig. 11 indicates the main source of energy for cooking in both villages. Results in Ambros were distributed as follows, firewood (70%), paraffin stove (59%) and gas stove (54%). In Maramanzhi, firewood accounted for 83%, followed by electric stoves (52%) and paraffin stoves (2%) as well as solar energy (2%).

## 4 Discussion

### 4.1 Socio-economic demographics

This study aimed to analyse household food security in Ambros village, located in Umzimvubu Local Municipality, Eastern Cape and Maramanzhi village, situated in the Musina Local Municipality of Limpopo province. Agricultural production and socio-economic attributes, such as age, gender, household size, education and income, play a significant role in understanding food security at a household level because these factors influence decisions related to food within a household [61]. The dominance of female-headed households in South Africa has been realised in previous research. Regrettably, these households are normally single-parent households with increased food security and poverty vulnerability [68]. The possible explanation for the older generation being more engaged in cultivation could be that the younger generation desires to study in urban areas or pursue other careers besides agriculture [[69], [70], [71]]. The study indicated a notable prevalence of household sizes between 1 and 3 in the study area. Other studies discovered that household size influences the food security status of households because an increase in household size reduces the probability of achieving food security within a household [72]. Analysis of income per month emphasizes that households in Ambros are characterised by low-income levels, with the majority reliant on social grants and earning less than R3 000 per month. The General Household Survey reveals that grants accounted for 61,4% of household income in Limpopo and 65,4% in the Eastern Cape, meaning grants are the primary source of income for households in these provinces [46]. Other studies have reported similar findings whereby social grants were the dominant source of income for the majority of the surveyed population and used as a strategy to protect against hunger and food insecurity [4,45,53,73]. While [74] found that social grants and cultivated land area were not correlated, our results suggest that this income had implications for cultivation because it restricted the expansion of the cultivated land area. Some household heads desired to cultivate or expand their fields or home gardens but were restricted by their low income, which is mainly used to purchase household food.

A greater proportion of household heads in Maramanzhi earned a higher income due to the significant contributions of agriculture and formal employment. This higher income from agriculture was supported by access to facilities for selling crops to reputable fresh produce markets. In contrast to Maramanzhi, market access was limited in Ambros. Several scholars lament that smallholder farmers in South Africa are confronted by market access challenges and high transaction costs for market participation because they live in remote areas defined by inadequate information, skills, transport, storage facilities and poorly maintained market infrastructure and roads [[75], [76], [77]]. Considering that smallholder farmers are confronted by several challenges that restrict their access to markets, government interventions need to develop the capacity of extension officers [69].

### 4.2 Household food security dynamics

Regarding food availability**,** a few similarities were noted in the type of crops cultivated in home gardens and fields in the study sites. Beetroot, pumpkins and peas were commonly cultivated in Ambros, while watermelon, butternut, muxe, nuts, morogo, okra, peri-peri and sweet potatoes were more prevalent in Maramanzhi. These variations can be ascribed to various underlying factors, prevalent in the two villages, including differences in food preferences, culture, climatic conditions, soil fertility and agricultural production patterns [78]. These variations are likely to continue because it is projected that Limpopo will experience a drier climate in the future. This means the type of cultivated crops will possibly also change [79]. Although a wider variety of crops were cultivated in Maramanzhi compared to Ambros; maize was common in both villages. These results resonate and concur with other studies which have reported that maize is a common crop cultivated by smallholder farmers in both the Eastern Cape and Limpopo province [80,81]. Despite the need for increased maize availability in the study area, both Ambros and Maramanzhi lie within zones that experienced the lowest maize productivity in 2020. Maize was also reported as the most required basic food for households (Fig. 9), suggesting it is an important staple food for rural households. Low agricultural productivity raises concerns because it impedes efforts to solve the economic, ecological and political challenges faced by rural livelihoods [82]. Other studies attest that maize products are a staple food in Limpopo [42] and Eastern Cape [15]. In most low-income households, food options are restricted but mainly consist of starch such as bread and maize, with few fruits and vegetables being consumed [16,58]. Thus, increased maize production can play a key role in enhancing food security for rural households [13].

In terms of food access, over half of the surveyed household heads in the study owned fields and/or gardens. This implies that these household heads had access to arable land that could be cultivated to supplement food for household consumption. These results align with [83] who suggest that in order to reduce the effects of resource shortages and inadequate agricultural support, households prefer to cultivate their fields and home gardens on an ongoing basis to improve food security. Despite most cultivation occurring during the period between 2016 and 2021, the decline in field and home garden cultivation was a critical challenge that also affected food security in the study area because a decline in the cultivated land area reduced available food for households. Previous research attests that deagrarianization processes compromise rural food security and sovereignty because food availability challenges intensify [84]. In other instances, studies have established a positive correlation between cultivated land area and food security because the area under cultivation influences profits from crops [15]. In line with these results [69], found a positive correlation between government agricultural assistance and sustainable crop production. The ownership of arable land, disaggregated by gender, indicated pronounced differences but fathers were the main people involved in cultivation at the household level. The contributions of both males and females play an important role in household food security and income because females mainly engage in subsistence farming, while males cultivate to earn an income to meet household needs [85].

There was a notable prevalence of food accessed through purchasing but household heads within the study area mainly preferred cultivated crops. The dependence on food purchases can be attributed to how crop production has various benefits but does not provide sufficient food for daily consumption [33]. Purchasing food is not sustainable for rural livelihoods because most households in this study area experienced ongoing food shortages and received low household income, which doesn't guarantee sufficient food. Although, purchasing food is a crucial strategy for ensuring food security, those with insufficient income become vulnerable to obtaining food of poor quality and low diversity [16]. Moreover, in rural communities of South Africa, households in remote areas (just like Ambros and Maramanzhi) are confronted by expensive local supplies and transport costs [86]. More household heads, particularly in Ambros lamented that cultivation reduced the money spent on food. Thus, home production needs to be encouraged as a strategy to not only supplement food purchases and reduce food access challenges but to also mitigate the effects of shocks such as increasing food prices. As highlighted by this study, food was rated as a very important reason for cultivating. This emphasizes the important role of cultivation for rural households and livelihoods. Compared to all other household expenses such as health care, education, farming and clothing, household heads spent a greater share of their income securing food demands. During times of food shortages households adopted various coping strategies to meet food demands. Coping strategies refer to methods of survival applied by households during unanticipated livelihood failures [63]. The majority of household heads in Ambros mostly rely on food loans from the supermarket, while those in Maramanzhi borrow money to purchase food. These results are in line with the findings from [53], who discovered that rural households confronted by food shortages adopt different coping strategies, including borrowing money for food. Neither Ambros nor Maramanzhi villages reported severely food-insecure households. However, difficulties in accessing sufficient food remain a prominent feature defining rural households because a considerable proportion of households were classified as mildly or moderately food insecure in both villages. Similar patterns were noticed in other studies that have applied various indicators, including HFIAS, in former homelands of South Africa. The results highlighted that the majority of households and smallholder farmers have a varied prevalence of food security statuses ranging from “food secure” [87] to “food secure to mildly food insecure” [73] and finally “severely food insecure” [4,42,88].

The utilization dimension highlighted that basic services, including water and electricity not only assist with food preparation but are also significant for crop production. Households in the study area were primarily reliant on communal taps but households in Ambros village still accessed water through rivers and dams, suggesting water is limited in this village. A similar pattern was noticed in the study of [78] who discovered that the province of Eastern Cape is one of the driest provinces with persistent challenges of water scarcity. This lack of adequate water supply demotivates smallholder farmers and compromises the prospect of sustainable agricultural production, thereby increasing food insecurity vulnerabilities. Firewood was reportedly the dominant source of cooking energy in both villages. However, this method is not sustainable as it emits greenhouse gases that contribute to climate change [89]. Furthermore, the use of electric stoves was not mentioned in Ambros, mainly because there was no electricity in the village during data collection. This absence hindered food security since food storage options became limited, forcing households to keep food for shorter periods. While fridge and cupboard storage methods are practiced in both Ambros and Maramanzhi, some households (particularly in Maramanzhi) have adopted indigenous methods, including drying fruit and vegetables and digging holes to store food for extended periods. Indigenous food management methods need to be reinforced at the household level so that food can be sustained at minimum costs [90].

### 4.3 Study limitations

Primary data used in this study was collected in 2021, thus it provides the food security status for the same year. More longitudinal studies are needed to understand changes in the status of household food security. This study focused on two villages only but showed granular details, thus other studies can focus on larger scales. This study analysed household food security in the context of deagrarianization in South Africa. Other studies could focus on food security in the context of various factors that include climate change, education statuses, employment and land reform. The results could further be disaggregated by factors that include gender, age and household size.

## 5 Recommendations and conclusion

This study recommends that agricultural development programmes invest more capital for agricultural development to allow for sustainable strategies that revive fallow fields and home gardens in former homelands of South Africa. This could reduce the reliance on purchased food and increase crop production and productivity for households. The government also needs to develop infrastructure such as roads and water supplies to assist with improving water supplies and, subsequently, crop production in rural localities such as Ambros, which continue to be affected by water shortages. Dissemination of information related to climate change, crop production methods, post-harvest storage, soil management and extension services is required to encourage cultivation, especially for the youth. In order to enhance crop production and productivity, people need to be educated about sustainable farming methods such as drought-resistant crops. Other interventions should focus on supporting smallholder farmers to participate in markets; for example, selling produce in groups could meet the quantities required by retail stores. This study has highlighted that deagrarianization coupled with low income intensifies food insecurity vulnerabilities because reductions in the area of cultivated fields or home gardens translate to fewer crops, lower food diversity and increased reliance on purchased food. These patterns raise concerns since the majority of households do not earn enough income to purchase sufficient food but have access to arable land which is not fully utilized for sustainable crop production. As a result, food access challenges remain prevalent. Although rural households rely on various coping strategies to buffer against hunger, household crop production can assist in mitigating household food insecurity challenges.

## CRediT authorship contribution statement

**Felicity Aphiwe Mkhongi:** Writing – review & editing, Writing – original draft, Visualization, Validation, Methodology, Investigation, Formal analysis, Conceptualization. **Walter Musakwa:** Writing – review & editing, Visualization, Supervision, Software, Methodology, Investigation, Formal analysis, Conceptualization. **Tholang Mokhele:** Writing – review & editing, Visualization, Supervision, Software, Methodology, Formal analysis, Conceptualization.

## Informed consent statement

All participants provided written informed consent to participate in the study and for their data to be published. This ensured that participants were informed about their voluntary participation and permission to withdraw their participation at any given time from the study.

## Data availability statement

The food security data used for this study is available on Mendeley Data:

https://data.mendeley.com/datasets/pxywtmxx26 under https://doi.org/10.17632/pxywtmxx26.1.

The Harvard Dataverse data is available on:

GGCP10: A Global Gridded Crop Production Dataset at 10km Resolution from 2010 to 2020 - Harvard Dataverse.

## Ethical approval

This study was reviewed and approved by the Ethics and Plagiarism Committee (FEPC) of the Faculty of Engineering and the Built Environment at the University of Johannesburg, with ethics approval reference UJ-FEBE_FEPC_00307 on the August 12, 2021.

## Declaration of competing interest

The authors declare that they have no known competing financial interests or personal relationships that could have appeared to influence the work reported in this paper.
