# Supplementary material for: Household food security contextualised: A comparison of Ambros and Maramanzhi villages, South Africa
Source: Heliyon. 2024 Oct 30;10(21):e39971. doi: 10.1016/j.heliyon.2024.e39971 (PMC11567040; doi:10.1016/j.heliyon.2024.e39971)
Supplement: Multimedia component 1 [file mmc1.docx]

**Dynamics of deagrarianization in the Eastern Cape and Limpopo provinces of South Africa: Questionnaire**

Name of interviewer: ___________________________________

Date and time: ________________________________________

Village name: _________________________________________

District:______________________________________________

Province:_____________________________________________

**Section A: Socio-economic demographics**

1. Gender

| Male | Female |
| --- | --- |
|  |  |

2. In which year were you born?

| Year |
| --- |
|  |

3. Who is living with you in this household?

| Partner |  |
| --- | --- |
| Children |  |
| Grandchildren |  |
| Relatives |  |
| Other |  |

4. Highest level of education completed

| 0= No formal education | 1= Primary | 2= Secondary | 3= Tertiary |
| --- | --- | --- | --- |
|  |  |  |  |

5. Household size

| 1-3 | 4-6 | 7-9 | 10+ |
| --- | --- | --- | --- |
|  |  |  |  |

6. Sources of income in household

| Agriculture | Social grant | Formal income | Remittances | Other |
| --- | --- | --- | --- | --- |
|  |  |  |  |  |

7. Total estimated household income per month

| <3000 | 3001-6000 | 6001-9000 | >9001 |
| --- | --- | --- | --- |
|  |  |  |  |

**Section B: Cultivation patterns, socio-economic and ecological driving forces of deagrarianization**

1a. Do you have a...

|  | Yes | No | Estimated size (H) |
| --- | --- | --- | --- |
| Field |  |  |  |
| Garden |  |  |  |

1b. If yes, when last did you cultivate your…(Explain)

|  | Year |
| --- | --- |
| Field |  |
| Garden |  |

1c. What do you plant in your…

| Field |  |
| --- | --- |
|  |  |
| Garden |  |
|  |  |

2. What is the main reason for cultivating? (rank 1 least important to 4 most important)

| Food provision for family |  |
| --- | --- |
| Income provision for family |  |
| For exercise |  |
| Other |  |

3. Who is mainly involved in crop cultivation in this household?

| Grandfather | Grandmother | Father | Mother | Child | Labourer | No one |
| --- | --- | --- | --- | --- | --- | --- |
|  |  |  |  |  |  |  |

4. What type of farmer are you?

| Eat with family | Eat with family and sell | Sell | Farmer who is not primarily dependent on produce |
| --- | --- | --- | --- |
|  |  |  |  |

5. Do you water the plants in your garden?

| Yes | No |
| --- | --- |
|  |  |

5a. If yes, water source used for your garden.

| Tap | Tank | Dam | River | Rain harvest |
| --- | --- | --- | --- | --- |
|  |  |  |  |  |

6. Do you water the plants in your field?

| Yes | No |
| --- | --- |
|  |  |

7a . If yes, water source used for your field.

| Tap | Tank | Dam | River | Rain harvest |
| --- | --- | --- | --- | --- |
|  |  |  |  |  |

8. What are the reasons for cultivation changes in your household?

**Section C: Food security**

9. Source of drinking water

| Communal Tap | Household Tap | Tank | Dam | River |
| --- | --- | --- | --- | --- |
|  |  |  |  |  |

10. How often do you buy food for household consumption?

| Weekly | Fortnight | Monthly |
| --- | --- | --- |
|  |  |  |

11. Household Food Insecurity Access Scale (HFIAS) Measurement Tool

| No. | Question | Response Option | Code |
| --- | --- | --- | --- |
| Q1. | In the past four weeks, did you worry that your household would not have enough food? | 0 = No (skip to Q2)  1=Yes |  |
| Q1a. | How often did this happen? | 1 = Rarely (once or twice in the past four weeks)  How often did this happen?  2 = Sometimes (three to ten times in the past four weeks)  3 = Often (more than ten times in the past four weeks) |  |
| Q2. | In the past four weeks, were you or any household member not able to eat the kinds of foods you preferred because of a lack of resources? | 0 = No (skip to Q3)  1=Yes |  |
| Q2a. | How often did this happen? | 1 = Rarely (once or twice in the past four weeks)  How often did this happen?  2 = Sometimes (three to ten times in the past four weeks)  3 = Often (more than ten times in the past four weeks) |  |
| Q3. | In the past four weeks, did you or any household member have to eat a limited variety of foods due to a lack of resources? | 0 = No (skip to Q4)  1 = Yes |  |
| Q3a. | How often did this happen? | 1 = Rarely (once or twice in the past four weeks)  How often did this happen?  2 = Sometimes (three to ten times in the past four weeks)  3 = Often (more than ten times in the past four weeks) |  |
| Q4. | In the past four weeks, did you or any household member have to eat some foods that you really did not want to eat because of a lack of resources to obtain other types of food? | 0 = No (skip to Q5)  1 = Yes |  |
| Q4a. | How often did this happen? | 1 = Rarely (once or twice in the past four weeks)  How often did this happen?  2 = Sometimes (three to ten times in the past four weeks)  3 = Often (more than ten times in the past four weeks) |  |
| Q5. | In the past four weeks, did you or any household member have to eat a smaller meal than you felt you needed because there was not enough food? | 0 = No (skip to Q6)  1 = Yes |  |
| Q5a. | How often did this happen? | 1 = Rarely (once or twice in the past four weeks)  How often did this happen?  2 = Sometimes (three to ten times in the past four weeks)  3 = Often (more than ten times in the past four weeks) |  |
| Q6. | In the past four weeks, did you or any other household member have to eat fewer meals in a day because there was not enough food? | 0 = No (skip to Q7)  1 = Yes |  |
| Q6a. | How often did this happen? | 1 = Rarely (once or twice in the past four weeks)  How often did this happen?  2 = Sometimes (three to ten times in the past four weeks)  3 = Often (more than ten times in the past four weeks) |  |
| Q7. | In the past four weeks, was there ever no food to eat of any kind in your household because of lack of resources to get food? | 0 = No (skip to Q8)  1 = Yes |  |
| Q7a. | How often did this happen? | 1 = Rarely (once or twice in the past four weeks)  How often did this happen?  2 = Sometimes (three to ten times in the past four weeks)  3 = Often (more than ten times in the past four weeks) |  |
| Q8. | In the past four weeks, did you or any household member go to sleep at night hungry because there was not enough food? | 0 = No (skip to Q9)  1 = Yes |  |
| Q8a. | How often did this happen? | 1 = Rarely (once or twice in the past four weeks)  How often did this happen?  2 = Sometimes (three to ten times in the past four weeks)  3 = Often (more than ten times in the past four weeks) |  |
| Q9. | In the past four weeks, did you or any household member go a whole day and night without eating anything because there was not enough food? | 0 = No (questionnaire is finished)  1 = Yes |  |
| Q9a. | How often did this happen? | 1 = Rarely (once or twice in the past four weeks)  How often did this happen?  2 = Sometimes (three to ten times in the past four weeks)  3 = Often (more than ten times in the past four weeks) |  |

12. What do you consider as a basic daily food requirement for your household?

13. How do you store your food?

14. Do transportation costs affect your food availability?

15. Approximately how many months of the year do you eat vegetables from your own garden and field?

16. How do you access food? (tick all that apply)

| Cultivation | Purchase | Donation |
| --- | --- | --- |
|  |  |  |

17. Do you prefer cultivating or purchasing crops? (explain)

18. What are the biggest expenses in this household? (rank-1 least to 5 most expense)

Food

Clothing

Farming

Education

Health Care

19. Does your income enable you to purchase all the food you need for the month?

| Never | Sometimes | Often | Always |
| --- | --- | --- | --- |
|  |  |  |  |

20. Does cultivation reduce your household money spent on food.

| Never | Sometimes | Often | Always |
| --- | --- | --- | --- |
|  |  |  |  |

21. Do you have access to selling your produce?

| Yes | No |
| --- | --- |
|  |  |

21a. If yes, who do you sell to?

22. Has purchasing made life easier or harder?

| Easier | Harder |
| --- | --- |
|  |  |

23. How do you prepare your food?

| Electric stove | Gas stove | Paraffin stove | Firewood | Solar energy |
| --- | --- | --- | --- | --- |
|  |  |  |  |  |
|  |  |  |  |  |

24. Do you prepare food according to nutritional value or not?

| 0=Yes | 1=No |
| --- | --- |
|  |  |

25. How many meals does your family have a day?

| 2 times | 3 times | 4 times | More than 4 times |
| --- | --- | --- | --- |
|  |  |  |  |

26. Do you suffer from food shortages if you do not cultivate?

| Strongly disagree | Disagree | Neutral | Agree | Strongly agree |
| --- | --- | --- | --- | --- |
|  |  |  |  |  |

27. How do you deal with food shortages?

| Eat less food | Borrow money | Loan food from supermarket | Ask relatives or neighbours |
| --- | --- | --- | --- |
|  |  |  |  |

28. Does subsistence production improve your food…

|  | Never | Sometimes | Often | Always |
| --- | --- | --- | --- | --- |
| Availability |  |  |  |  |
| Accessibility |  |  |  |  |
| Utilization |  |  |  |  |
| Stability |  |  |  |  |

**Thank you for participating in this study**
